# 

**Published:** 1890-04-19

**Authors:** 


### NEW OFFICES IN THE STRAND.

The Hospital *is now published at* **140, Strand, W.C.** *It has been found desirable to establish* The Hospital *in this central and convenient locality, owing to the rapid extension of the business of this paper of late.*

### NOTICE TO CORRESPONDENTS.

All MS., Letters, Books for Review, and other matters intended for the Editor should be addressed The Editor, The Lodge, Porchester Square, London, W.

The Editor cannot undertake to return rejected M.S., even when accompanied by stamped directed envelope.

**Notice to Advertisers and Subscribers.**

All Advertisements, Orders for Copies of The Hospital, and Business Communications should be addressed to The Publisher, *not to the Editor*, The Hospital, 140, Strand, W.C.

**Bound Volumes of "The Hospital."**

Vol. VI., containing full page portraits of T.R.H. the Prince and Princess of Wales, and Miss Florence Nightingale, is obtainable at 4s., post free.

Orders will be received for Vol. VII., 4s. post free. Cases for binding may be had of the Publisher, at 1s. 6d. each.

**To Residents, Secretaries, and Matrons.**

The Editor will be glad to have the Regulations and each Report as issued by Asylums, Hospitals, Convalescent and Nursing Institutions, as well as those of other Charities, and an early copy in duplicate of all Appeals and Festival Notices, etc., addressed to *The Editor, The Lodge, Porchester Square, London, W.* Any superintendent, secretary, matron, or other chief official who regularly sends such information may be registered, on application to the Publisher, to receive free of cost a cover for binding The Hospital in half-yearly volumes.

### Scraps and Gleanings.

Harwich Cottage Hospital treated 82 patients last year. Funds are satisfactory.

The College of Physicians have banished *The Monthly Homœopathic Review* from their library.

Dr. B. W. Richardson spoke in favour of temperance to the National Teachers' Union last week.

The British steamer, Fulford, is in quarantine at Bordeaux, with two cases of cholera on board.

At Lisbon there are 234 medical practitioners, 141 pharmacists, 107 midwives, and only 13 dentists.

The Paris doctors believe that fasting men use an alkaloid of kola, of which a few drops go a very long way.

Cushendall Cottage Hospital issues a satisfactory report at the close of the fourth year of its existence.

Mdlle. Huet having tried to lecture against vivisection at Paris, was howled down by a mob of medical students.

The Royal Hospital for Diseases of the Chest benefited by an excellent dramatic entertainment given at the Albert Hall on Thursday.

Sir Henry Loch, the Governor of Cape Town, laid the foundation stone of the new leper hospital on Robben Island on the 8th inst.

Viscount Cross will preside at the annual meeting of the Hospital for Women and Children, Vincent Square, Westminster, on April 30.

Shrewsbury Hospital Saturday Fund has been divided as follows: Eye and Ear Hospital, £40; General Hospital, £21; Dispensary, £12.

There seems some fear of Mr. Blundell Maple's generous offer of building an infectious hospital at St. Albans being lost for want of a site.

Prince Doria, whose death, while under an anæsthetic, was announced the other day, has left three million lire (£120,000) to various charitable objects.

At the quarterly court of Addenbrooke's Hospital the chairman called attention to the liberal allowance of patients' tickets given in return for subscriptions.

Application must be made by the end of April for the three scholarships of the Grocers' Company, for medical research into the cause and prevention of any important disease.

A case of poisoning by phosphorus has taken place at Bedlington, in a miner's daughter, aged twenty-one, who confessed to having used matches when in a state of mental depression.

The Northern Technical and Recreative Institute has received a donation of £250 through the Islington branch of the London and Westminster Bank, from Mr. John Proctor, of Highbury.

A man who had been living in a most sordid manner in a garret in Paris for many years, was found dead in his room on a heap of filthy rags. Under the rags was discovered no less a sum than 100,000 francs.

Manchester still maintains its unenviable position at the head of the death-rate list of great towns. For the week ending April 5th, the death-rate in Manchester was 28·6, in Huddersfield 10·5, in London 16·7.

The Duke of Fife, K.T., has consented to open the East Marylebone Free Library on Thursday, May 1, at three p.m. Of the £1,000 required for its establishment, £930 has been already subscribed, including a donation of £500 from Lady Howard de Walden.

The grand fancy bazaar in aid of the funds of University College Hospital, to be held in the grounds of University College, Gower Street, W.C., will be opened by the Duchess of Fife on Thursday, June 5, at twelve noon, instead of June 4, as previously announced.

Madame Rosa Kerschbaum, who has taken the degree of doctor of medicine at a Swiss University, has just been authorized by special Imperial decree to conduct a hospital for eye diseases at Salzburg. This is the first case of a lady physician being admitted to medical practice in Austria.

The Duke of Westminster has written a letter to the secretary of the Manchester Cremation Society, conveying his best wishes for the complete success of the proposed company for the erection of a Crematorium in the neighbourhood of Manchester. He has also taken 100 shares in the company.

In consequence of the death of a boy, named John Kelly, from hydrophobia at Newtownards, the Lord Lieutenant of Ireland has directed the resident magistrate for the County of Down to convene a special meeting of county magistrates to consider steps for the prevention of the spread of rabies.

The Queen has sent a donation of £50 towards the funds of the City of London Pension Society, in response to an appeal by Mr. Sheriff Harris, who is to preside at the coming festival dinner on May 16 in aid of that well-known charity, which was founded by her Majesty's father, the Duke of Kent, in 1818.

The subject of the housing of the working classes will be dealt with by Lord Compton, at the annual meeting of the East London Church Fund, at the Mansion House, on April 21, and the Bishop of Bedford has been invited to form a committee to watch the question throughout the East London Ecclesiastical district.

At Worcester, Edward Davies, a vendor of quack medicines, has been sentenced to a month's hard labour, for causing a man named Reece, to swallow a liquid, thereby doing grevious bodily harm. Reece bought a bottle of stuff sold as a specific for colds, and swallowed the liquid, which proved to be a strong solution of ammonia.

Under the will of the late Sir James Tyler, the Merchant Taylors' Company, it is stated, will receive in trust for charitable purposes a most munificent sum. It is probable that the bequest will be devoted to the enlargement of the company's convalescent home. The London Missionary Society and the British and Foreign Bible Society also come in for handsome legacies.

Stafford Hospital Saturday Committee have passed the following resolution: "That we, the Hospital Saturday committee, object to the rule now existing on recommendation papers which enquires into the earnings of applicants, and respectfully ask the governors to withdraw the rule, and the committee suspends the collection until some more satisfactory arrangements are made."

